# Internal supravesical hernia as a rare cauase of intestinal obstruction: a case report

**DOI:** 10.1186/1752-1947-3-9333

**Published:** 2009-12-16

**Authors:** Mamadou Cissé, Ibrahima Konaté, Ousmane Ka, Madieng Dieng, Abdarahmane Dia, Cheikh T Touré

**Affiliations:** 1 Clinique Chirurgicale, Hôpital Aristide Le Dantec, Avenue Pasteur, BP 3001, Dakar, Sénégal

## Abstract

**Introduction:**

Supravesical hernias develop at the supravesical fossa between the remnants of the urachus and the left or right umbilical artery. They are often the cause of intestinal obstruction. We describe the anatomical variant of the supravesical hernia in this case and discuss the pre-operative findings revealed by computed tomography. We discuss diagnostic and therapeutic procedures, and review other anatomical variants.

**Case presentation:**

A 60-year-old Senegalese man was admitted with a two-day history of small bowel obstruction. A physical examination showed abdominal distension. An abdominal X-ray revealed dilated small bowel loops. A computed tomography scan showed an image at the left iliac fossa that suggested an intussusception. A median laparotomy showed a left lateral internal supravesical hernia. The hernia was reduced and the defect was closed. The patient recovered uneventfully.

**Conclusions:**

Supravesical hernia is a possible cause of intestinal obstruction and diagnosis is very often made intraoperatively. Morphological examinations, such as computed tomography scanning, can lead to a preoperative diagnosis. Laparoscopy may be useful for diagnosis and therapy.

## Introduction

Supravesical hernias develop at the supravesical fossa between the remnants of the urachus and the left or right umbilical artery. They have many anatomical variants and are often the cause of intestinal obstruction. A preoperative diagnosis is unusual despite the use of investigations such as computed tomography (CT). We report a case of a left lateral supravesical variety revealed by intestinal obstruction. We review the anatomical variants of supravesical hernias and discuss the diagnostic and therapeutic procedures involved in their management.

## Case presentation

**Figure 1:**
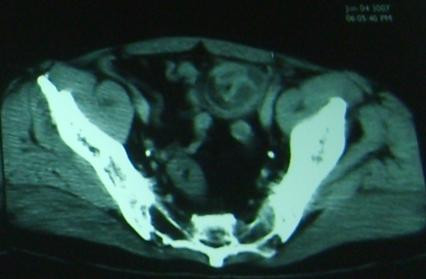
**A computed tomography scan showing an image (arrow) in the left iliac fossa that suggests an intussusception**.

**Figure 2:**
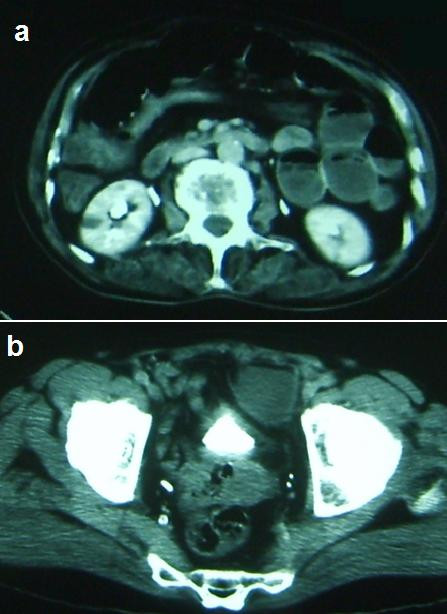
**A computed tomography scan showing a distended loop with transitional zone**.

A 60-year-old Senegalese man with no relevant medical history was admitted in June 2007 with a two-day history of small bowel obstruction characterized by abdominal pain and vomiting. On examination, the patient was found in good general condition with a pulse rate of 90/min, a blood pressure of 100/60 mmHg, and a temperature of 37.5°C. Physical and examination showed that the patient had abdominal distension without any peritoneal signs. Rectal examination was normal. An uncomplicated inguinal hernia was also found. The patient's renal function and other blood tests were all normal. An abdominal X-ray revealed dilated small bowel loops. A CT scan showed an image in the patient's left iliac fossa that suggested an intussusception (Figure 1). A distension of intestinal loops (Figure 2) with transitional zone was also found.

After preoperative resuscitation, a median laparotomy was performed on the patient. The exploration showed a left lateral supravesical internal hernia with an incarcerated viable ileal loop. The hernia was then reduced by cautious traction and the defect was closed with 1/0 polyester interrupted stitches. The patient recovered uneventfully.

## Discussion

The supravesical fossa is the abdominal wall area between the remnants of the urachus (median umbilical ligament) and the left or right umbilical artery (medial umbilical ligament) [1,2]. The remnant of the urachus divides into the right and left fossa. There are two variants of supravesical hernias: an external form caused by the laxity of the vesical preperitoneal tissue, and an internal one with a growing hernia sac from back to front and above the bladder in a sagittal paramedian direction [1,3]. External supravesical hernia often occurs as a direct inguinal hernia. Except in specific cases of post-hernia surgery, supravesical hernias are almost always acquired and sometimes associated with inguinal hernias, as in our patient [1,3,4].

Skandalakis *et al*. proposed the simpler terms "anterior supravesical", "right or left lateral supravesical", and "posterior supravesical" depending on whether the hernia passed in front of, beside, or behind the bladder, respectively [4].

Diagnosis of these supravesical hernias, including that in our patient's case, is almost always made intraoperatively. A preoperative diagnosis is very unusual. In some cases, a CT scan may suggest the diagnosis by showing the herniated loop so near the bladder that it actually distorts the wall [1,4]. Magnetic resonance imaging (MRI) and cystoscopy may also help in preoperative diagnosis [1]. Therapy is surgical and its objective is to reduce the herniated viscera and then suture the orifice. The excision of the hernial sac is an unnecessary procedure. As some authors have reported, these procedures can be done via laparoscopy [5,6].

## Conclusions

Supravesical hernias are rare but potential causes of intestinal obstruction due to the confinement of loops in the supravesical fossa. The diagnosis is often made intraoperatively. Some morphological examinations such as a CT scan can lead to a preoperative diagnosis. Laparoscopy may also be used for diagnosis and for therapy.

## Consent

Written informed consent was obtained from the patient for the publication of this case report and any accompanying images. A copy of the written consent is available for review by the Editor-in-Chief of this journal.

## Competing interests

The authors declare that they have no competing interests.

## Authors' contributions

MC performed the surgical procedure and reported the case. IK and OK interpreted and analysed the tomodensitometry findings. MD participated in the diagnostic and therapeutic decisions. AD and CT made major contributions in writing the manuscript. All authors read and approved the final manuscript.
